# RiskRadar: development and pilot results of a technical intervention targeting combination prevention regarding HIV, viral hepatitis, sexually transmitted infections and tuberculosis

**DOI:** 10.1186/s12879-021-06501-0

**Published:** 2021-09-13

**Authors:** Christine Kakalou, Eleftheria Polychronidou, Vicky Drosou, Vlasios K. Dimitriadis, Thomas Dermaris, Rafael Kordonias, Aris Papaprodromou, Triantafillos Tsirelis, Christos Maramis, Konstantinos Votis, Dimitrios Tzovaras, Domenico Savarino, Manuel Maffeo, Nedim Jasic, Tatjana Nemeth-Blažić, Zoran Dominković, Dubravko Pogledić, Iva Jovovic, Agne Simkunaite-Zazecke, Loreta Stoniene, Antonella Sammut, Lella Cosmaro, Pantelis Natsiavas

**Affiliations:** 1 Institute of Applied Biosciences, Centre for Research and Technology Hellas, Thermi, Thessaloniki, Greece; 2 Information Technologies Institute, Centre for Research and Technology Hellas, Thermi, Thessaloniki, Greece; 3 Fondazione LILA Milano - Italian League for Fighting AIDS, Milan, Italy; 4 Arcigay - Associazione LGBTI Italiana, Bologna, Italy; 5 Croce Rossa Italiana, Rome, Italy; 6 CIPH Croatian Institute of Public Health, Zagreb, Croatia; 7 Iskorak, Zagreb, Croatia; 8 Life Quality Improvement Organisation “Flight”, Zagreb, Croatia; 9 ULAC/CCDA Centre for Communicable Diseases and AIDS, Vilnius, Lithuania; 10 RPLC Republican Center for Addictive Disorders, Vilnius, Lithuania; 11 Public Mental Health Services. Ministry for Health, Valletta, Malta

**Keywords:** Human Immunodeficiency Viruses (HIV), Integrated approach, Combination prevention, eHealth, Risk assessment

## Abstract

**Background:**

The HIV pandemic impacts the lives of millions and despite the global coordinated response, innovative actions are still needed to end it. A major challenge is the added burden of coinfections such as viral hepatitis, tuberculosis and various sexually transmitted infections in terms of prevention, treatment and increased morbidity in individuals with HIV infection. A need for combination prevention strategies, tailored to high-risk key populations arises and technology-based interventions can be a valuable asset. The COVID-19 pandemic challenged the delivery of existing services and added stress to existing public health and clinical structures but also highlighted the potential of exploiting technical solutions for interventions regarding infectious diseases. In this paper we report the design process, results and evaluation findings from the pilots of ‘RiskRadar’—a web and mobile application aiming to support combination prevention, testing and linkage to care for HIV, viral hepatitis, various sexually transmitted infections and tuberculosis.

**Methods:**

RiskRadar was developed for the INTEGRATE Joint Action’s aim to improve, adapt and pilot innovative digital tools for combination prevention. RiskRadar was designed iteratively using informed end-user-oriented approaches. Emphasis was placed on the Risk Calculator that enables users to assess their risk of exposure to one or more of the four disease areas, make informed decisions to seek testing or care and adjust their behaviours ultimately aiming to harm/risk reduction. RiskRadar has been piloted in three countries, namely Croatia, Italy and Lithuania.

**Results:**

RiskRadar has been used 1347 times across all platforms so far. More than 90% of users have found RiskRadar useful and would use it again, especially the Risk Calculator component. Almost 49.25% are men and 29.85% are in the age group of 25–34. The application has scored 5.2/7 in the User Experience Questionnaire, where it is mainly described as “supportive” and “easy-to-use”. The qualitative evaluation of RiskRadar also yielded positive feedback.

**Conclusions:**

Pilot results demonstrate above average satisfaction with RiskRadar and high user-reported usability scores, supporting the idea that technical interventions could significantly support combination prevention actions on Sexually Transmitted Infections.

## Background

HIV, viral hepatitis, Tuberculosis (TB) and other Sexually Transmitted Infections (STIs) continue to pose major public health challenges globally and gaps in the coverage of the continuum of care services are widely acknowledged [1, 2]. The term “combination prevention” refers to approaches that promote a combination of biomedical, behavioral, and structural interventions adapted to the needs of specific communities [3].

However, one-size-fits-all approaches have failed to support the broad and complex spectrum of HIV, viral hepatitis and STIs prevention needs [4]; Effective prevention strategies for the different key populations most at risk [5] (i.e. Men who have sex with Men (MSM), People Who Inject Drugs (PWID), transgender people, sex workers, people living in prisons or other closed settings and migrants) indicate that interventions designed for specific populations and social contexts have enhanced the adoption of prevention behaviours and the uptake of care services [6–9]. To this end, a number of studies exploring the effectiveness and acceptability of such efforts show encouraging results regarding the uptake and actionable takeaways from Information and Communication Technologies (ICT)-assisted interventions [10–14]. However, it should be emphasized that, regardless of how innovative a technical intervention might seem, it must also take social issues into account [15].

The INTEGRATE Joint Action (JA) [16] has implemented integrated activities to improve awareness, prevention, early diagnosis and linkage to care for HIV, viral hepatitis, STIs and TB in Europe. It aimed to bring together ICT experts with infectious disease scientists, clinicians, civil society and members of academia to design, pilot and assess the effectiveness of a new ‘INTEGRATE ICT Tool’, i.e. the RiskRadar (RR). This web and mobile application offers basic information on the four disease areas and tools for finding testing sites across Europe, notifying sexual or needle sharing partners as well as for self-assessing protective behaviours to prevent HIV infection, hepatitis, other STIs and TB. In this paper we describe the design process and report on the results of the RiskRadar pilot test conducted in Croatia, Italy and Lithuania during the second half of 2020.

## Methods

**Fig. 1:**
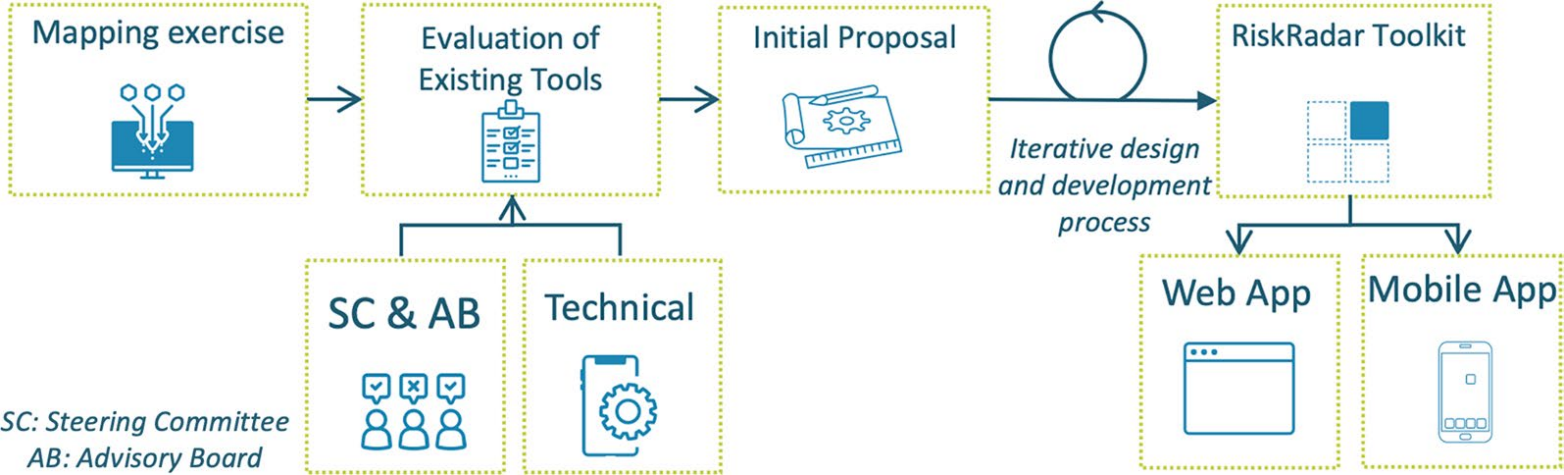
RiskRadar design and development methodology

The RiskRadar application design process (see Fig. 1) began with a mapping exercise of existing ICT tools for prevention, testing and linkage to care for four disease areas, i.e. HIV, viral hepatitis, TB and STIs. 115 ICT tools were initially proposed for evaluation; the list was then reduced to 53 tools and ordered by topic (Prevention, Harm Reduction, PrEP, Testing, Partner Notification (PN), Linkage to Care). INTEGRATE’s Steering Committee (SC) and Advisory Board (AB) members rated the mapping matrix while a technical overview was also conducted to assess adaptability, reproducibility, data relevance of the selection. Since effectiveness studies on the tools under review were sparse [17], the evaluation was based on the expertise of the various stakeholders and their extensive experience utilising such tools in their workflow. Several tools were considered for adaptation (see Table 1) due to features providing added value to the toolkit under development. “YOUR ENDING HIV TOOLKIT” offered a comprehensive suite of tools, “Chemsex Care Plan” contained insights on harm reduction and non-judgmental language, “PrEP in Europe” provided the most up-to-date information about PrEP while “PrEP Locator” pointed to PrEP providers (albeit in the USA). “What’s your number” was proposed as a base for the Risk Calculator and “Let Them Know” and “Don’t Spread It” introduced anonymous Partner Notification via SMS and/or email.

**Table 1:** Shortlisted tools for HIV, viral hepatitis, STIs or TB as rated by the mapping exercise

ICT Tool Name	Technical evaluation *Scale 1–5*			Review by SC/AB and INTEGRATE-involved organisations *Scale 1–5*
	Adaptable	Reproducible	Data relevance	
YOUR ENDINGHIV TOOLKIT	4	5	4	4
Chemsex Care Plan	5	5	5	4
PrEP in Europe	5	5	5	3
PrEP Locator	5	5	0	3
What's your number	4	5	2	2
Let Them Know	5	5	4	4
Don't Spread It	5	5	4	4

These shortlisted tools drove the initial proposal of the RiskRadar structure; the adaptation and integration process aimed to accommodate all four disease areas and the pilot-specific target audiences, while considering the pre-existing tools’ strengths, weaknesses and perceived effectiveness to address the different information and prevention aspects. The overall technical design of RiskRadar was based on informed User eXperience (UX), end-user oriented design and communication approaches [18–22]. The following priorities were defined: (a) understandable and adaptable content to fit diverse target groups; (b) acceptable, non-judgemental, non-threatening and non-fear-inducing language and imagery; (c) privacy and confidentiality along GDPR, and (d) emphasis on user empowerment by exploiting insights of user perspectives as contributed by the pilot partners involved, in an iterative design and development process; All involved stakeholders (mainly NGOs and patient organisations) examined RiskRadar’s design and offered valuable points towards the inclusion of specific group needs, such as promoting PrEP and Hepatitis B vaccination to MSM, providing Hepatitis A&C vaccination advice to migrants, including a lightweight web application option to be accessed at various low-threshold points for PWID without smartphones etc. This “feedback loop” was repeated for each version of RiskRadar until a consensus was reached in a final stakeholders meeting.

The RiskRadar components can be summarized as following: (a) *Factsheets* (FS) offering basic information about the four disease areas (a TB infographic is presented individually to highlight the differences to the other three), (b) *Risk Calculator* (RC) that helps users assess their risk of exposure to the different diseases according to their behaviour and to make informed decisions in order to adjust their protective behaviours, (c) *PrEP* and *Undetectable = Untransmittable* (U=U) information, (d) *Test Finder* (TF) with filtering capabilities per disease and country, (e) *Partner Notification* (PN), an anonymous, non-traceable SMS service to notify one’s partners that can be accessed via a unique random code (in QR form for smartphones and in serial form for web users) that only diagnosed patients receive from an authorized healthcare professional, (f) *Reminders* (only featured in the mobile app) for the users with an estimated risky behaviour to get tested every 6 months that can be disabled at any time, and (g) *Evaluation Questionnaire* (EQ) [23] to collect information regarding end-user satisfaction and perceived impact. For the most challenging components, the RC and PN, a Data Protection Impact Assessment was conducted to identify and mitigate any risk in terms of information security.

**Fig. 2:**
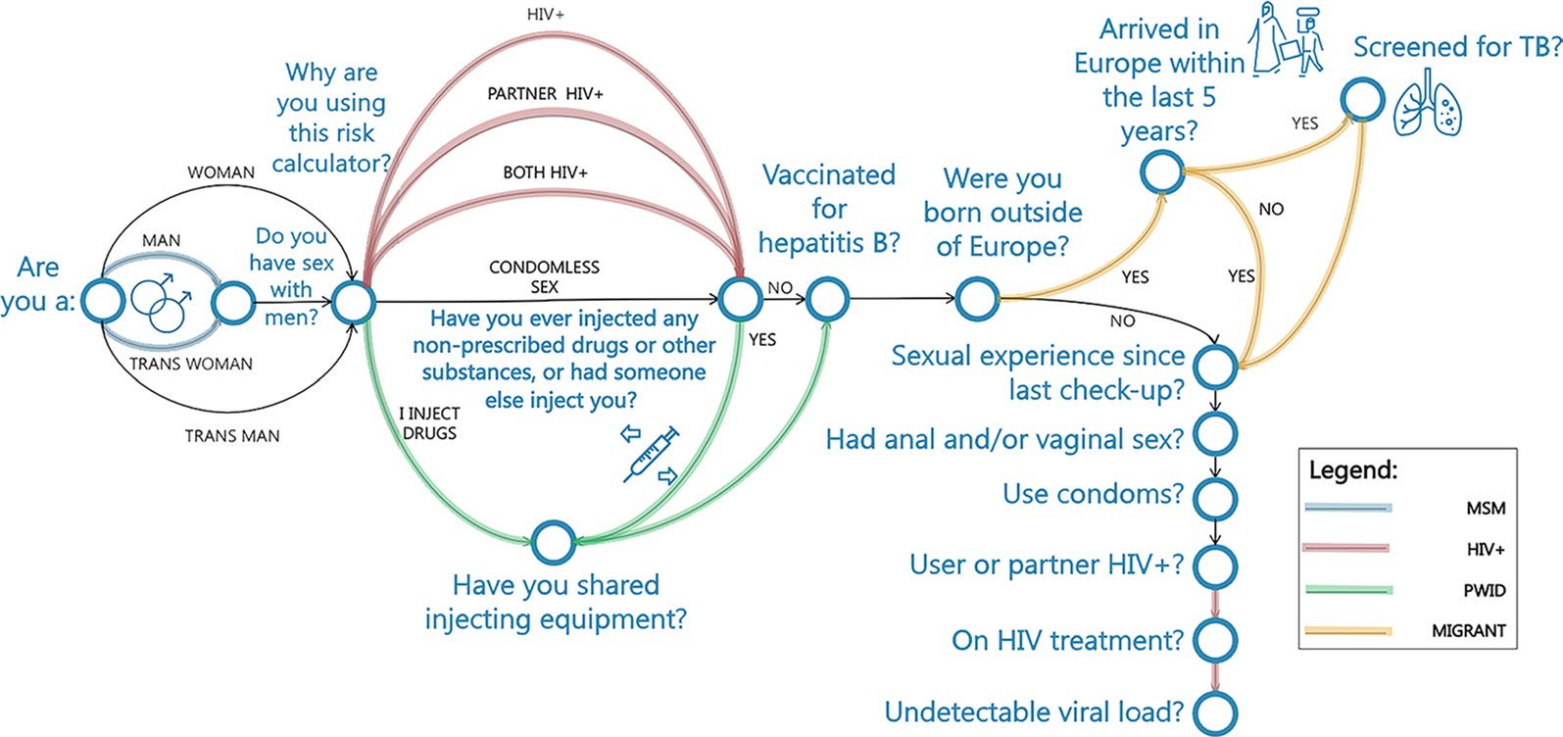
Decision tree for the Risk Calculator

The functionality of the Risk Calculator component aimed to facilitate the individual risk assessment based on the decision trees (a) described in WHO and ECDC guidelines [24–26] and (b) derived from the analysis of existing single-disease risk assessment tools for HIV, hepatitis or STIs considered during the mapping exercise. To this end, various risk factors were identified through 16 questions that follow conditional logic (see Table 2) while upon the Risk Calculator’s completion, a comprehensive answer is presented, depicting the estimated risk overall and per risk factor. Additionally, if the user reports condom use, they can opt to answer 3 further questions to get advice on how to select the correct condom type. The final decision tree (see Fig. 2) is based on input from a wide range of infectious disease scientists, clinicians and civil society members participating in the project, through an iterative design process aiming to balance the sufficient granularity of answers while ensuring user retention. Vital risk factors resulting from the users’ answers were selected to be stored anonymously in RiskRadar’s secure database for further analysis. Other factors (e.g. PrEP use) drove the conditional logic of the Risk Calculator component as well, but were not stored.

**Table 2:** Risk assessment factors for the Risk Calculator Module

Risk factor	Possible answer
HIV status	Positive/Negative/Unknown
Partner’s HIV status	Positive/Negative/Unknown
Condomless sex incidence	Yes/No
MSM	Yes/No
Immigration status (Is user an immigrant?)	Yes/No
Person Who Injects Drugs (PWID)	Yes/No
Person Who Injects Drugs (PWID)—sharing injecting equipment	Yes/No
Person Who Injects Drugs (PWID)—not sharing injecting equipment	Yes/No
Hepatitis B vaccination status	Vaccinated/Unvaccinated/Unknown
Tuberculosis vaccination status (only if user is immigrant)	Vaccinated/Unvaccinated /Unknown
Condom Test: fit of the condom	Just Right/Loose/Short/Tight
Condom Test: latex allergy	Yes/No
Condom: adequate sensation	Yes/No

**Fig. 3:**
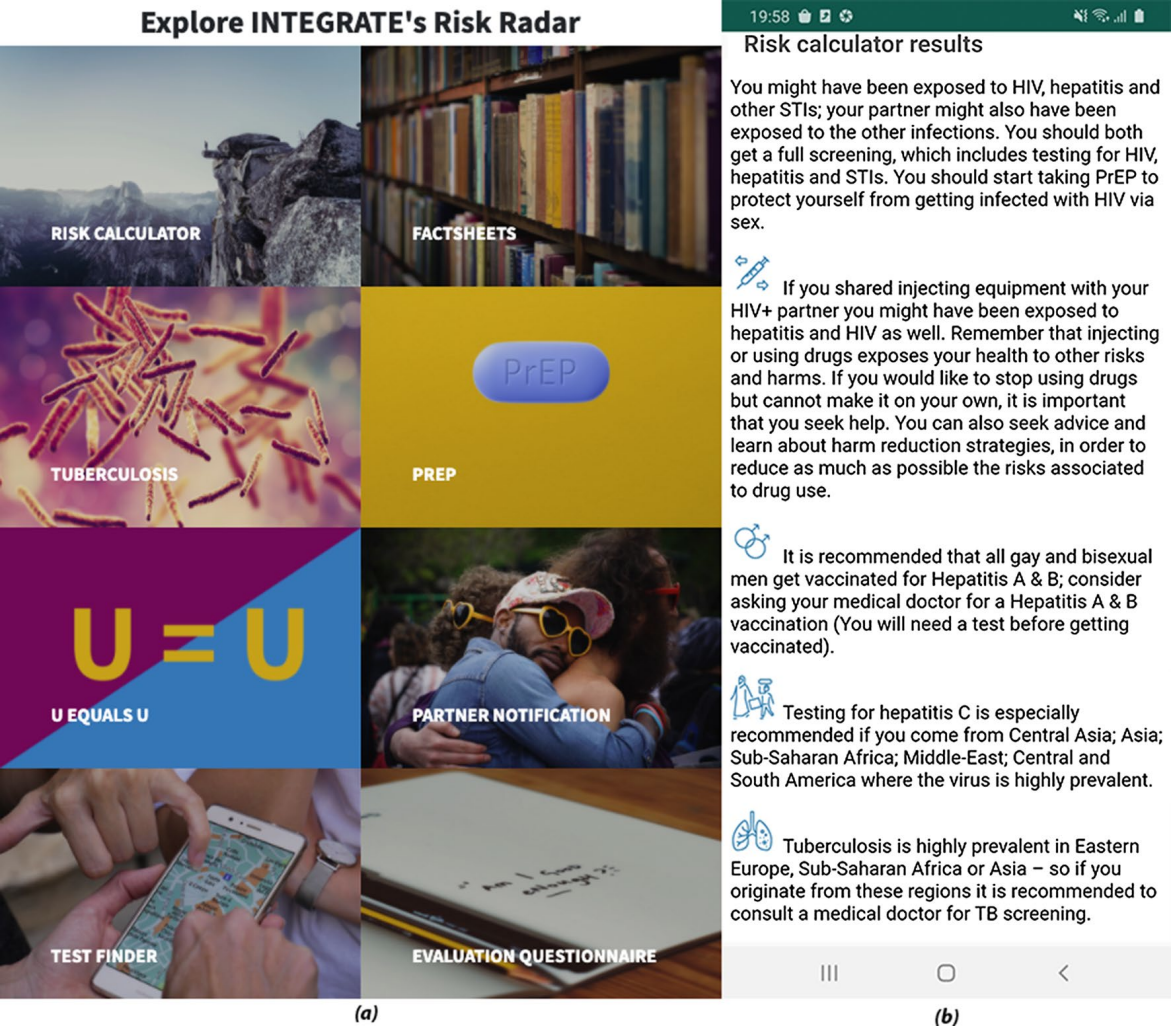
**a** RiskRadar's components (web application view) and **b** Risk Calculator answer (mobile app view)

RiskRadar is available via a web application [27], an Android mobile app [28] and an iOS based app [29] for Apple devices ecosystem (iPhones, iPads etc.). It was translated in the native languages and piloted during the second half of 2020 in Croatia, Italy and Lithuania, targeting different key populations to evaluate the application’s impact in diverse settings. RiskRadar’s various components (see Fig. 3 part a) were evaluated concerning their overall usability and acceptability per country; The respective metrics include usage statistics from Google Analytics, Google Play and the Apple Store for the web, Android and iOS apps respectively, risk factor metrics from the Risk Calculator component and the answers to the evaluation questionnaires submitted via the RiskRadar toolkit. Finally, a qualitative evaluation was conducted in Italy and Lithuania, in the form of semi-structured online interviews and face-to-face focus groups. The participants were introduced to a validation scenario describing the basic actions to be performed while using the RiskRadar, aiming to solicit reactions and thoughts during the hands-on inspection of the toolkit. The full User Experience Questionnaire [23] was completed at the end of those sessions to measure the users’ satisfaction.

Regarding promotional activities, RiskRadar banners and articles have been featured on pilot and affiliated partner’s websites and social media accounts (including targeted social media paid advertisements) and on Italy’s and Lithuania’s Ministry of Health websites. RiskRadar has been presented in multiple occasions at national and regional meetings to public health specialists and other involved stakeholders. Targeted promotion was handled by each organisation as needed, using available communication channels and through their day-to-day interactions with potential end-users. To this end, paid campaigns were launched on Grindr, a prominent social networking app for gay, bi, trans and queer people in Italy, and on the gay.it website. Moreover, the toolkit was presented to people accessing low-threshold service locations where it could gain visibility among vulnerable risk groups.

## Results

RiskRadar was officially launched on July 6th, 2020 -after some months’ delay due to the COVID19 pandemic; the piloting phase lasted until December 31st, 2020. In total, it was accessed 817 times via web, while 270 and 260 users have downloaded the app on Android and iOS respectively. The Risk Calculator component (see Fig. 3 part b), one of the main attractions of RiskRadar as a whole, has been used 1106 times.

In terms of adoption during the pilot period, most users were attracted in Italy (Table 3). The web application shows an average session duration of 14 min and 51 s while only 9.70% of users navigate away from the landing page without exploring the application further; both indicators denote a high retention and interest of visitors in RiskRadar. Regarding localisation in general, it should be noted that country information for each web application visit or mobile download is deducted by the user-selected language. In terms of privacy, no location data are retrieved by the user’s browser or mobile device, in accordance to GDPR. Consequently, the English version metrics contain data that are impossible to attribute correctly to any pilot country. Furthermore, the Lithuanian language isn’t readily available for the native iOS app -only as an option after the initial English installation- so all Lithuanian Apple ecosystem devices are included in the metrics for the English version.

**Table 3:** RiskRadar usage per pilot country

Platform	Total visits/downloads	Croatia	Italy	Lithuania	English version^a^ (available in all countries)
Web application	817	13.21%	25.45%	8.81%	52.51%
Mobile app (Android and iOS)	530	4.33%	73.77%	5.28%	16.60%^b^
Total	1347	9.72%	44.47%	7.42%	38.38%

^a^Localisation data extrapolated from the user’s language selection – any user from a pilot country viewing the English version is not included in the region metrics

^b^Lithuanian language not available for native iOS apps. The Lithuanian version is available only after language selection in the downloaded English version and this affects region metrics

Tables 4 and 5 present the aggregated results (overall and per language) collected through the Risk Calculator component to gain insights on population groups using this particular module and by extension, the target audiences of the RiskRadar pilots. As expected, MSM constitute a sizable portion of the user base, reaching up to 43.50% in Italy where the pilot focused on MSM and migrants. 6.33% reported being a migrant and a joint percentage of 3.53% answered that they were using injectable drugs. The condom test was only taken 47 times, with 38.29% reporting that condoms fit too tightly, 21.28% noticing irritation due to latex and 19.15% complaining about diminished/inadequate feeling with condom use. It should be noted that users may choose to repeat the Risk Calculator test to explore risky behaviours, however due to privacy issues, the application did not keep information to identify unique users. Although this process obviously lacks accuracy, there is nevertheless an opportunity to extract some comparative insights per country. Also, although users may change their answers on risky behaviours, some basic user categorization is expected to be consistent such as sexual preference, immigration status etc. More accurate information regarding the targeted populations and intended actions would be gathered by the piloting organisations who provide linkage to testing and care but they were cancelled due to the pandemic.

**Table 4:** Risk factors recorded by users' answers in the RC (HIV status, sex-related risks)

Language	Total RC tests taken	MSM	Both user and partner(s) are HIV+	User is HIV+	User’s partner(s) is/are HIV+	Had unprotected sex
All languages	1106	27.67%	0.72%	5.70%	22.15%	14.47%
English^a^	394	13.96%	1.02%	3.55%	12.69%	7.11%
Italian	446	43.50%	0.67%	9.42%	34.08%	25.11%
Croatian	157	31.21%	0.64%	3.18%	20.38%	10.19%
Lithuanian	109	7.34%	0.00%	1.83%	10.09%	3.67%

^*a*^Localisation data extrapolated from the user’s language selection—any user from a pilot country viewing the English version is not included in the region metrics

**Table 5:** Risk factors recorded by users' answers in the RC (Drug injection risk, vaccination and immigrant status)

Language	Total RC Tests Taken	PWID (Not sharing injecting materials)	PWID (Sharing injecting materials)	Not vaccinated for hepatitis B	Migrant	Has not had a TB screening (only for migrants)
All languages	1106	1.45%	2.08%	19.44%	6.33%	1.08%
English^a^	394	1.27%	2.54%	9.14%	7.61%	2.03%
Italian	446	2.02%	2.24%	28.70%	8.97%	0.90%
Croatian	157	0.00%	1.27%	19.11%	0.00%	0.00%
Lithuanian	109	1.83%	0.92%	19.27%	0.00%	0.00%

^a^Localisation data extrapolated from the user’s language selection – any user from a pilot country viewing the English version is not included in the region metrics

**Fig. 4:**
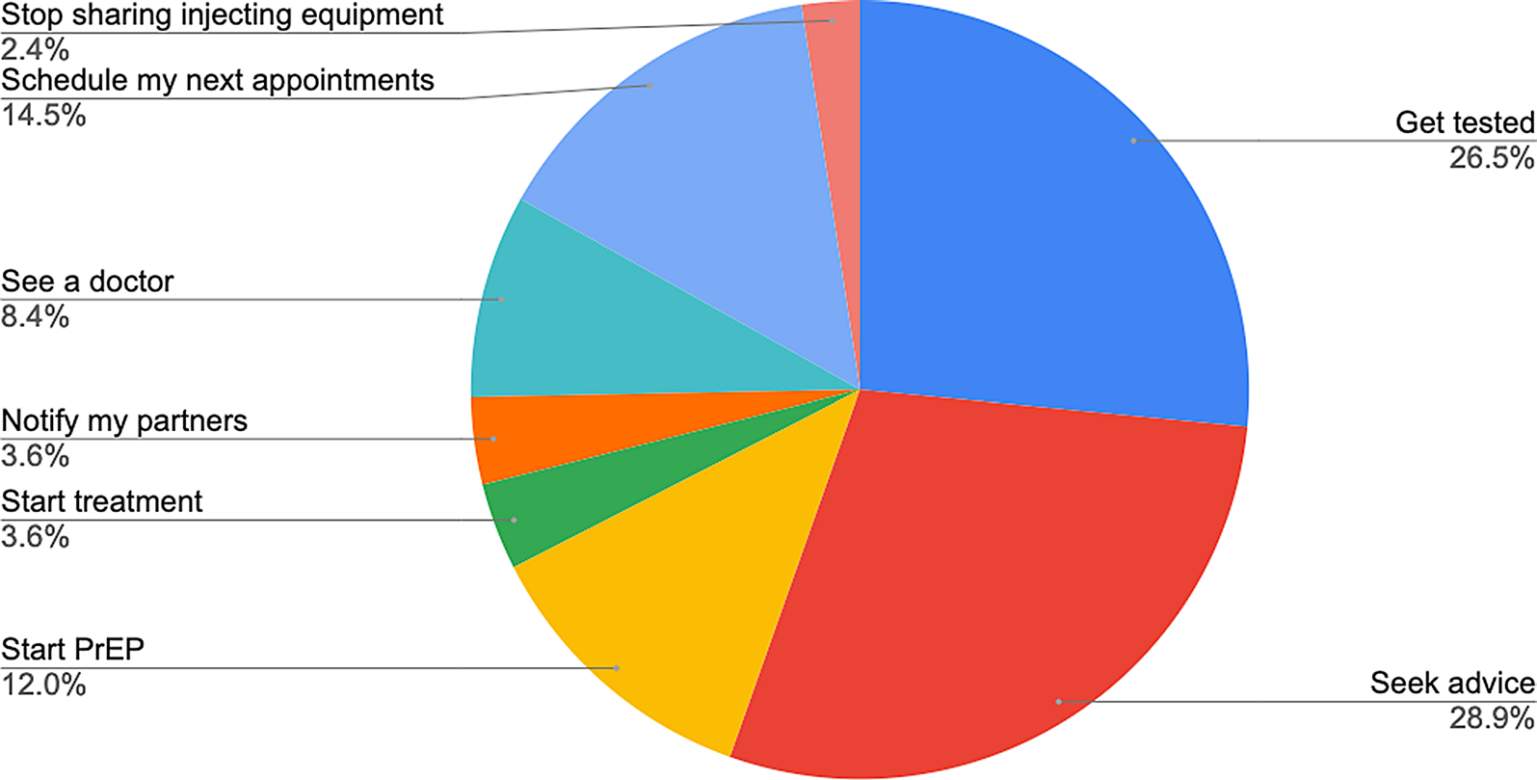
Action taken after RiskRadar use

**Fig. 5:**
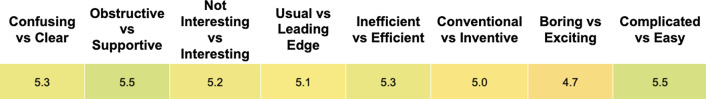
Average scores for the main 8 aspects of User Experience Questionnaire reported in-app

**Fig. 6:**
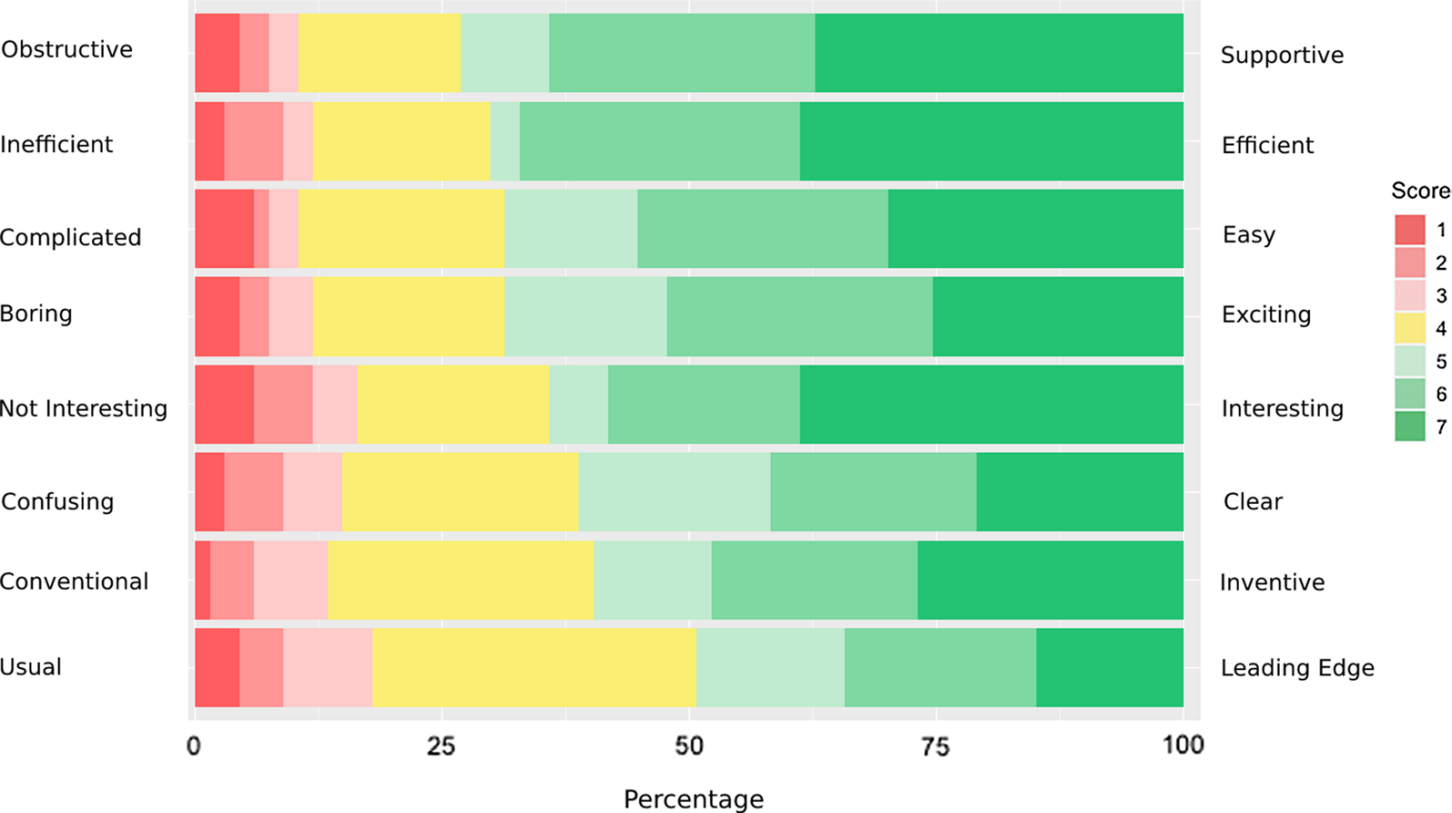
Likert rating scale results regarding the RiskRadar User Experience reported in-app

To evaluate RiskRadar in a systematic manner, a special component was developed containing (a) questions about its perceived usefulness and (b) a short user experience evaluation questionnaire (UEQ) [23]. This component, designed to be as unobtrusive as possible, was completed 67 times which corresponds to 4.97% of total visits, an acceptable response for this kind of in-app surveys [30]. Overall, 92.53% of responses found RiskRadar useful; 85.07% were positive to using RiskRadar again in the future. The various STIs Factsheets were ranked first regarding end-users’ interest (67.16%), followed by the Risk Calculator (64.18%), the Test Finder (38.80%) and U=U and PrEP information (41.79% and 40.29% respectively). Interestingly, 76.11% of responses indicated further action upon consulting the toolkit, mainly by seeking further advice (35.83%) and getting tested (32.83%) as seen in Fig. 4. Concerning the demographics, 49.25%, 20.89% and 2.99% of end-users identified as men, women and trans women respectively. 29.85% were in the 25–34 age group followed by 16.41% of users aged 35–44. RiskRadar achieved an average user experience score of 5.2 (with 7 being the highest possible score) and the highest ranked characteristic of the RiskRadar experience was support and ease-of-use (5.5/7) while the lowest was the excitement and inventiveness aspect (see Figs. 5 and  6).

**Fig. 7:**
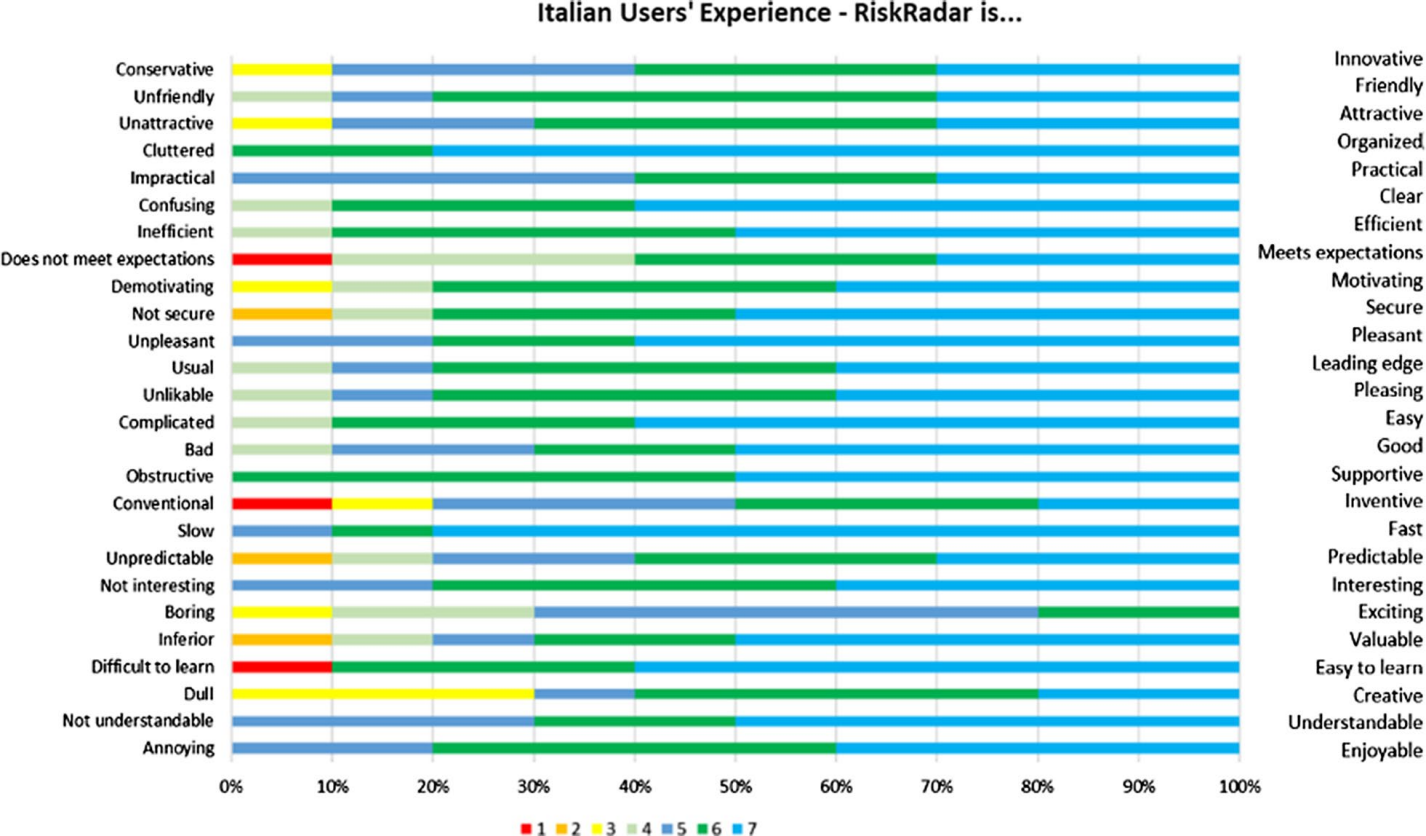
Likert rating scale results from the User Experience reported in the Italian evaluation

Regarding the qualitative evaluation, in Italy 10 participants were interviewed online by Italian pilot partners; 80.00% were men (Italian partners address mainly MSM) and 40.00% of them belonged in the 45–54 age range followed by the 35–44 and over 75 age range (20.00% each). Self-reported computer skills were average and above. Figure 7 presents the user experience scores where RiskRadar is rated as understandable, enjoyable, good, easy, efficient, clear, organized and friendly by all respondents, while it scores lower regarding creativity and inventiveness. All respondents appreciated the integration aspect of the tool.

**Fig. 8:**
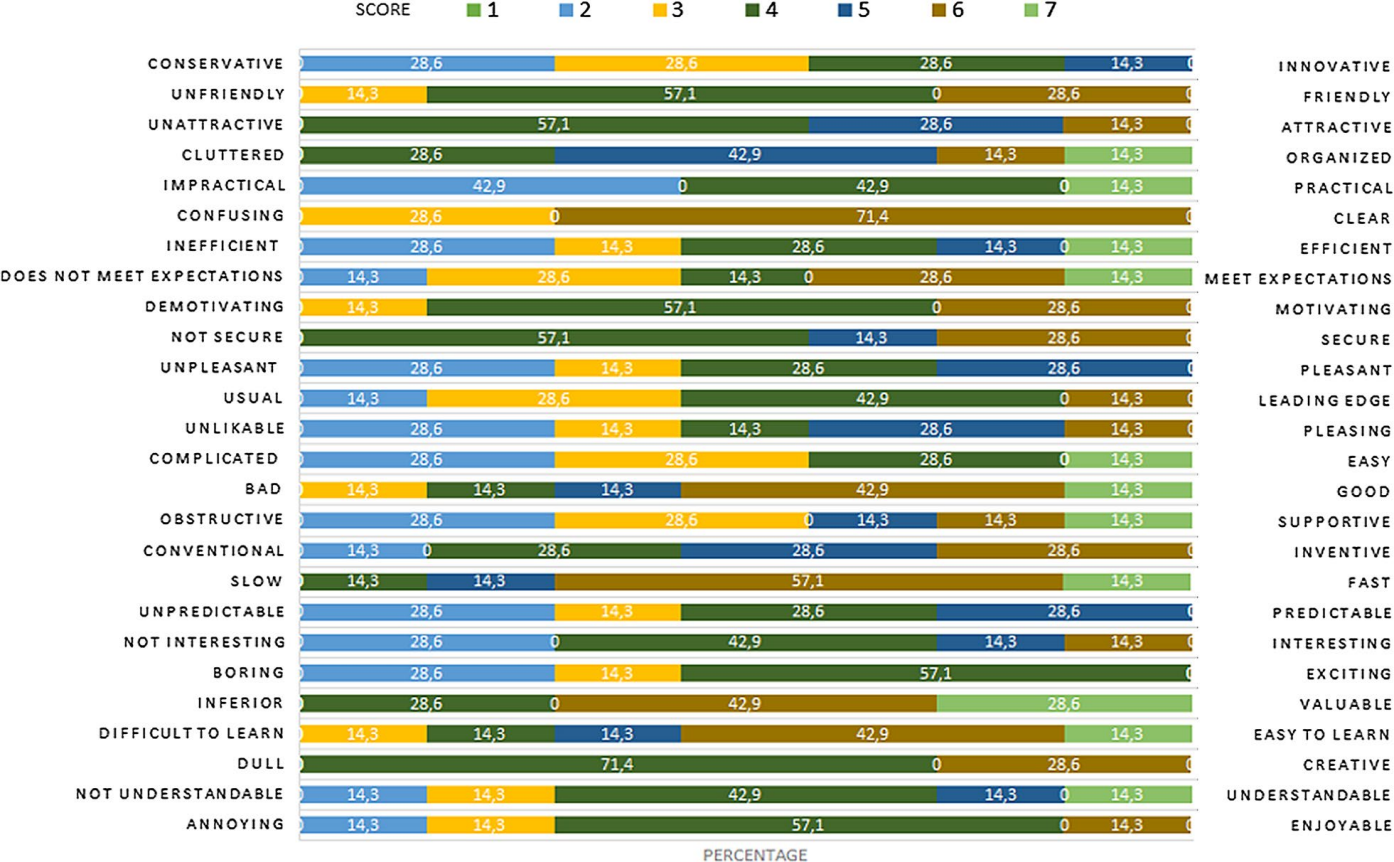
Likert rating scale results from the User Experience reported in the Lithuanian evaluation

In Lithuania, face to face focus groups were organised instead, involving 12 patients with diagnosed addictive disorders, however only 7 of them completed the UEQ after the meetings. All respondents were males; 71.40% belonged in the 35–44 age range and 28.60% in the 45–54 range. 6 participants self-assessed as having “bad” or “moderate” computer skills. Overall, the tool received positive feedback (see Fig. 8) however uptake of the tool was challenging in participants with insufficient computer literacy, in which case assistance and support was deemed necessary.

Finally, it should be noted that majority of the feedback received in the form of free-text comments and interviews focused on expanding the toolkit towards harm reduction advice, self-testing and PrEP services that are becoming more popular across the EU.

## Discussion

The development of RiskRadar exploited input from experts and stakeholders across the INTEGRATE JA to achieve the additive and synergistic effects of various combination prevention approaches, targeting certain vulnerable population groups. RiskRadar aims to accommodate various target audiences, countries and regions, disease areas accompanied by a broad scope of prevention, testing, empowerment, treatment and capacity building strategies. The developed application offers significantly increased levels of security, privacy and confidentiality that enhances its acceptability and impact on a wide audience in the EU.

RiskRadar’s development encountered numerous challenges, especially concerning the integration of TB-oriented artefacts due to the differences in the routes of transmission, prevention and care compared with the other diseases. Moreover, the coordination of information and translations across the 3 pilot countries, bearing in mind the particularities of each country’s settings, capacity and target populations, was far from trivial, especially during the COVID-19 pandemic. The Risk Calculator component proved to be the most challenging, since the integrated risk assessment algorithm for all 4 disease areas had to be created by prioritising risk based on each specific target group’s characteristics and the messages communicated by the RC answers required broad stakeholder input.

Regarding the limitations of this study, an inherent user selection bias should be noted. The INTEGRATE consortium designed the pilots aspiring to reach as wide an audience as possible and utilise any channels at their disposal, starting with already established networks (patients/clients already visiting NGO and low threshold sites, actively involved members of the civil society etc.). In the future, a controlled study engaging a representative sample of the targeted populations could provide more robust results. Furthermore, low computer literacy, prevalent in some vulnerable groups, has been identified as key factor which might hinder the uptake of similar interventions. Moreover, it should be highlighted that as COVID-19 pandemic affects most key populations disproportionately by hindering or at least delaying access to testing and treatment, especially for vulnerable populations [31, 32] and adding strain to the healthcare systems of low and middle income countries, where TB is endemic and patients may also be co-infected with HIV and other STIs [33]. To this end, COVID-19 significantly hindered the pilot roll out of RiskRadar, limiting the pilot application range and the potential impact of the intervention as a whole, as for example the ability to direct users to local organizations for continuity of care was restricted to traditional phone/email communications showcased in RiskRadar’s dedicated “Contact” section. On the other hand, it should be noted that digital tools have the potential to address some of the imposed challenges and to mitigate some of the added burden [31, 34–36];

As a whole, we consider RiskRadar’s uptake to be positive, taking into account the difficulties introduced by the COVID-19 pandemic, especially regarding the dissemination of the tool among the target groups. The majority of users in the pilots originate from Italy, partly due to the timely and coordinated efforts of the partners and the experience and traction gained from previous similar campaigns, but also because of the disparities in population size. All pilot countries managed to intensify their efforts during the last months while targeting key population groups and supporting their use of RiskRadar, which was clearly shown in the daily breakdown of usage metrics.

The overall evaluation of the tool was satisfactory with RiskRadar’s understandability and user friendliness scores being rated as highly positive. Furthermore, INTEGRATE’s Scientific Committee and Advisory Board members have suggested the additional expansion of the RiskRadar components to other combination prevention issues such as HIV and STIs self-tests, chemsex, harm reduction advice and services etc.

Outlining future work paths, RiskRadar’s further development should focus on (a) following an adaptive model to address the variety of needs per population group, (b) systematic evaluation and (c) long-term sustainability plans to maximise its impact.

## Conclusion

RiskRadar targets population groups at risk for HIV, hepatitis, STIs and TB with clear and simple combination prevention messages through several modular core components. Based on the overall experience of the RiskRadar development, we argue that RiskRadar and similar interventions have the potential to provide support towards combination prevention efforts, provided that considerable resources to ensure its accuracy, reliability, relevance and usefulness are invested in the future. As the COVID-19 pandemic has also clearly highlighted, the need for timely monitoring, prevention, testing and risk assessment constitute a necessity that ICT technologies can significantly address.

## Data Availability

Not applicable.

## Abbreviations

AB: Advisory Board
COVID-19: Coronavirus disease
ECDC: European Centre for Disease Prevention and Control
EU: European Union
GDPR: General Data Protection Regulation
HIV: Human Immunodeficiency Viruses
ICT: Information and Communications Technology
JA: Joint Action
MSM: Men who have Sex with Men
NGO: Non-Governmental Organisation
OS: Operating System
PN: Partner Notification
PrEP: Pre-Exposure Prophylaxis
PWID: Person Who Injects Drugs
RC: Risk Calculator
RR: Risk Radar
SC: Steering Committee
STIs: Sexually Transmitted Infections
TB: Tuberculosis
U=U: Undetectable = Untransmittable
UEQ: User Experience Questionnaire
UI: User Interface
UX: User Experience
WHO: World Health Organization
